# Rock mass classification prediction model using heuristic algorithms and support vector machines: a case study of Chambishi copper mine

**DOI:** 10.1038/s41598-022-05027-y

**Published:** 2022-01-18

**Authors:** Jianhua Hu, Tan Zhou, Shaowei Ma, Dongjie Yang, Mengmeng Guo, Pengli Huang

**Affiliations:** School of Resources and Safety Engineering, Central South University, Changsha, 410083 Hunan China

**Keywords:** Computational science, Petrology

## Abstract

The rock mass is one of the key parameters in engineering design. Accurate rock mass classification is also essential to ensure operational safety. Over the past decades, various models have been proposed to evaluate and predict rock mass. Among these models, artificial intelligence (AI) based models are becoming more popular due to their outstanding prediction results and generalization ability for multiinfluential factors. In order to develop an easy-to-use rock mass classification model, support vector machine (SVM) techniques are adopted as the basic prediction tools, and three types of optimization algorithms, i.e., particle swarm optimization (PSO), genetic algorithm (GA) and grey wolf optimization (GWO), are implemented to improve the prediction classification and optimize the hyper-parameters. A database was assembled, consisting of 80 sets of real engineering data, involving four influencing factors. The three combined models are compared in accuracy, precision, recall, F_1_ value and computational time. The results reveal that among three models, the GWO-SVC-based model shows the best classification performance by training. The accuracy of training and testing sets of GWO-SVC are 90.6250% (58/64) and 93.7500% (15/16), respectively. For Grades I, II, III, IV and V, the precision value is 1, 0.93, 0.90, 0.92, 0.83, the recall value is 1, 1, 0.93, 0.73, 0.83, and the F_1_ value is 1, 0.96, 0.92, 0.81, 0.83, respectively. Sensitivity analysis is performed to understand the influence of input parameters on rock mass classification. It shows that the sensitive factor in rock mass quality is the RQD. Finally, the GWO-SVC is employed to assess the quality of rocks from the southeastern ore body of the Chambishi copper mine. Overall, the current study demonstrates the potential of using artificial intelligence methods in rock mass assessment, rendering far better results than the previous reports.

## Introduction

The rock mass is a concrete manifestation of the non-linear coupling of multiple factors in complex rock systems, which is directly related to the selection of construction design parameters and overall safety. The accurate assessment of rock mass quality reflects the physical and mechanical properties of the rock mass and provides reliable bases for engineering stability analysis, disaster prediction, prevention and control^1^. Therefore, it is necessary to develop appropriate methods to predict and evaluate the quality of rock mass^2^.

Numerous studies have been performed for the assessment of rock mass quality. For instance, Terzaghi’s rock-load classification scheme can be considered as the first empirical rock mass classification system^3^. Thereafter, various other evaluation methods have also been proposed based on different engineering practices. For example, the classical single-index grading methods, such as the Protodyakonov coefficient *f* grading method, the tensile strength R_t_ gradin g method, the compressive strength R_c_ grading method^4^, Deer's RQD grading method^5^, and the elastic wave velocity Vp method^6^. In addition, there are grading methods with juxtaposition of indicators such as Chinese engineering rock grading standard (BQ method)^7^. With the development of systems engineering, the influence of multiple factors is considered in the assessment of rock quality. Bieniawski et al. have utilized the sum-difference method to integrate different factors and construct an RMR rock grading system^8^. Similarly, Barton et al. have employed the product method to establish the rock tunneling index (Q) grading system^9^. Naithani et al. used a Q-system for tunnel rock quality grading, which supported the choice of support method^10^. Subsequently, several other rock classification systems were built on this basis. For example, Laubscher combined RMR values and adjustment parameters for different factors under mining conditions to build MRMR systems^11^. The M-RMR system as developed by Unal is also based on the RMR system and includes additional features for the characterization of weak, stratifified, anisotropic and clay bearing rock masses^12^. As the study progresses, the utilization of fuzzy theory has significantly improved the generalization performance and accuracy of classification methods^2^. Daftaribesheli constructed the M-SMR system using the mamdani fuzzy algorithm and the SMR system, which quantifies the fuzziness in the rock system^13^. Chen et al. have proposed a classification method for the quality of surrounding rocks based on hierarchical analysis and fuzzy Delphi method^14^. Zhou et al. have improved the classification distinction by the grey clustering method, which enhanced the applicability of the classification method and improved the positive assessment rate of rock mass quality^15^. Hu et al. have established the RS-TOPSIS model and applied it for the mass classification of rock masses in underground engineering^16^. Zhou et al. have effectively combined experimental expertise and multidimensionality of rock masses by improving RES and the uncertainty cloud theory to obtain a novel assessment method, which does tendentious evaluation^17^. Nowadays, big data and artificial intelligence are developed rapidly and neural network models, based on prior knowledge, are proposed and applied in the geotechnical fields. Feng and Wang have described a novel approach to predict probable rock bursts in underground openings based on learning and adaptive recognition of neural networks^18^. Alimoradi et al. learned Tunnel Seismic Prediction (TSP-203) data by ANN model. The trained ANN successfully predicted the poorer geological regions in the tunnel^19^. Klose et al. learned six seismic features by Self-Organizing Mapping (SOM) model to describe the complex relationship between geological conditions and seismic parameters. The results show that the trained SOM model can predict the geological conditions well from the seismic monitoring data^20^. Jalalifar et al. used the fuzzyneural inference system and predicted RMR-value. They used three types of fuzzy-neural networks and showed that the subtractive clustering method is more efficient in predicting RMR-value^21^. Rad et al. successfully implemented the prediction of the RMR system output values by coupling the Chaos-ANFIS model^22^. More pertinent work about rock mass classification prediction using AI methods is tabulated in Table 1.

**Table 1:** Previous work about rock mass quality prediction using AI techniques.

Reference	Technique	Input	Output	Performance
Liu et al.^23^	SA-BPNN	Th, Tor, PR, RPM	UCS, DPW, α	MAPE
Liu et al.^24^	SST-SVR	Th, Tor, RPM	UCS, DPW, α	MSPE, R^2^
Mutlu et al.^25^	DF-HFIS	UCS, RQD, spacing of discontinuities, conditions of discontinuity, GW,	RMR	RMSE, R^2^, VAF
Hou et al.^26^	RF	TBM operation parameters	Rock mass classification	Accuracy
Barzegar et al. ^27^	SVM	n, Rn, Vp	UCS	R^2^
	ANFIS			
	SFL			
	MLP			
Asheghi et al.^28^	ICA-GFFN	Is, R_c_, γ, n, Vp, w	UCS	R^2^
	MLP			
	RBF			
	GFFN			
Jalalifar et al.^29^	ANFIS	UCS, RQD, Js, Jc, GW	RMR	RMSE, MAPE, VAF R^2^

*Th* Thrust, *Tor* Torque, *RPM* revolutions per minutes, *UCS* Uniaxial compressive strength, *DPW* Distance between planes of weakness, *α* orientation of discontinuities, *RQD* Rock quality designation, *RMR* Rock mass rating, *GW* Groundwater condition, *Js* Joint spacing, *Jc* Joint condition, *Vp* P-wave velocity, *n%* Porosity, *Rn* Rebound hardness, *Is* point load index, γ Density, *w* Water absorption, *MAPE* Mean absolute percentage error, *MSPE* Mean squared percentage error, *R*^*2*^ Coefficient of determination, *RMSE* Root mean square error, *VAF* Variance accounted for.

These studies have improved the theory and methods of rock mass grading to a certain extent. For instance, the traditional single-indicator or multi-indicator comprehensive evaluation method is easy to operate, but the way is idealized and does not match with the actual complex rock system. The majority of models are unpopular and have been restricted to specific geological environments or countries. On the other hand, the selection of factor levels and weights in the fuzzy mathematical theory is a challenging task and different models may result in different classification results, limiting the generalization performance of the proposed model. And third, the mining of relevant data is difficult when AI-based methods are used to assess rock mass quality, limiting the accuracy of the proposed model. At the same time, traditional methods such as neural networks are less capable of learning small sample data, and the trained models are prone to extreme cases such as poor accuracy or overfitting. Hence, based on a large number of rock mass classification results, it is necessary to organize existing cases, establish a rock mass quality database and train an efficient rock mass classification model based on a small sample classification algorithm.

Zheng et al. established a small database containing 80 sets of tunnel rock samples and conducted a study on tunnel rock mass classification based on the SVM algorithm, which verified the excellent small sample learning ability of the SVM algorithm^30^. It has many attractive properties such as a strong mathematical foundation, few tuning parameters, fast classification and high generalization capability. The success of SVM relies on the selection of key parameters (c, g). In general, the machine learning models, without combining optimisation algorithms, are inefficient^31^. The traditional SVMs are often paired with the traversal method, such as the grid search (GS) method, to perform optimal computations. The traversal method is computationally intensive and renders low accuracy. Fayed propose a new method to prune the data by removing those data points that have a very small chance of becoming support vectors to ensure shorter search time and the acquisition of globally optimal parameters^32^. A disadvantage of the methods is that they are highly sensitive to the initial parameters. If they are far from the global optimal solution, they often converge to a local optimal solution^33^.

Recently, heuristic algorithm have been widely used and considered to have a big chance to converge to the global optimum^34^. Ren and Bai too offered twin methodologies for constraint refinement in SVM: particle swarm optimization PSO-SVM and GA-SVM^35^. Zhou et al. coupled the GA algorithm and PSO algorithm for parameter selection to reduce the parameter finding time and improve the accuracy^36^. Hussein used the simulated annealing (SA) algorithm to find the parameters of the SVM and validated it using data from the UCI machine learning repository, experimentally verifying that the accuracy of the SA-SVM algorithm is greater than that of the SVM algorithm^37^. Li established GA-SVR model, PSO-SVR model, and salp swarm algorithm SSA-SVR model, and used the models to predict fber-reinforced CPB strength, and the study showed that the heuristic algorithm can capture the hyperparameters of SVR model better than the grid search algorithm^38^. In geotechnical engineering, Arsalan et al. developed an SVR model to successfully obtain dynamic RQD values of rock mass during tunnel excavation^39^. Li uses the uckoo search algorithm-improved support vector machine method is applied to slope stability analysis and parameter inversion, which proves the advantages of hybrid heuristic algorithm in parameter optimization^40^. The above study proves that the SVM algorithm meets the needs of small sample rock mass classification. The heuristic algorithm can better balance the state of global search and local search and effectively escape from local optimum. The combination of heuristic algorithm and SVM can better exploit the advantages of SVM algorithm. Meanwhile to the best knowledge of the authors, currently, the application of heuristic algorithm combined with SVM in improving the performance of machine learning models for rock mass classification has not been reported.

This paper will focus on the development and application of support vector machine in rock mass classification, and try to study the heuristic algorithms in the support vector machine parameter optimization, algorithm improvement of the application, and MATLAB as a programming platform, to achieve the method. The main work of this study is as follows:

- In this study, we collected 80 groups of rock mass quality datasets from different studies to build a database to improve the problem of small and single-source data sets in previous studies.
- The support vector machine (SVM) models are utilized as the main classification tools combined with three optimization algorithms, i.e. GA, PSO, grey wolf optimization (GWO) to find key SVM parameters (c, g). Meanwhile, cross-validations are employed to examine the classification capability of different models.
- Five mathematical indices, i.e. accuracy, precision, recall, F_1_ value, and computational time, are used to assess the classification performance. The sensitivity analysis is implemented to understand the sensitivity of each input parameter on rock mass quality grade.
- Finally, the trained model is used for rock mass quality grading of the southeast ore body of the Chambishi copper mine.

## Support vector machines and heuristic algorithms

### Support vector machines (SVM)

#### Support vector classification model (SVC)

**Figure 1:**
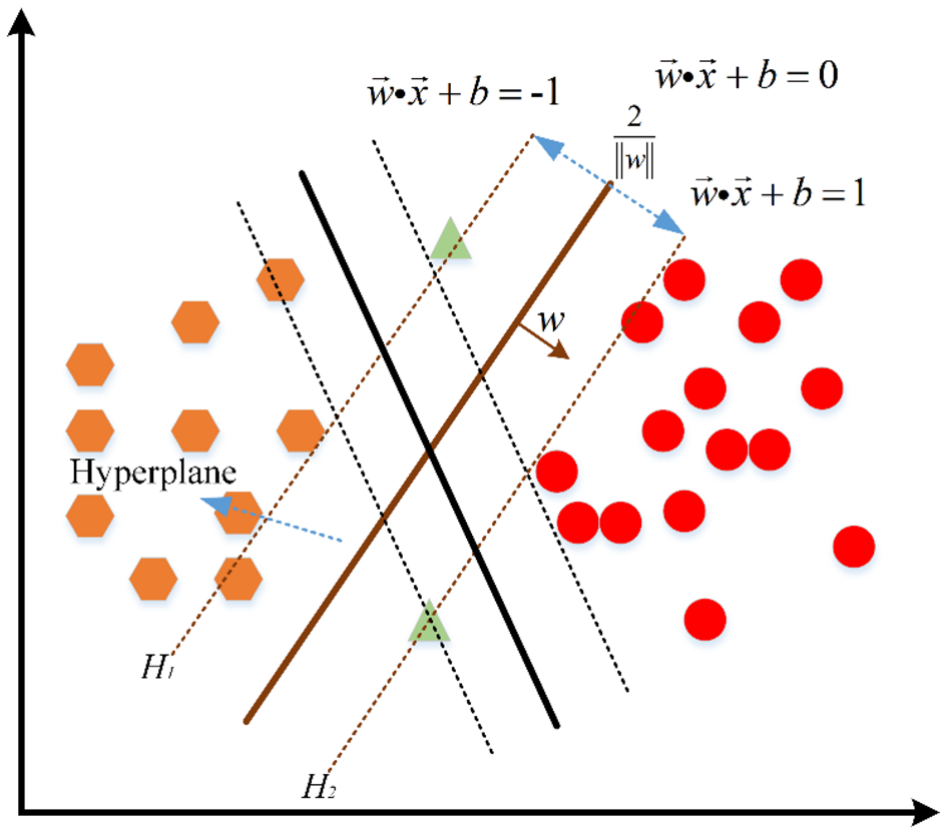
Schematic illustration of the SVC classification algorithm.

SVM is a statistical learning method based on Vapnik–Chervonenkis theory (VC) and structural risk minimization principle of the statistical learning theory^41^. SVM mainly learns, classifies and predicts the small datasets. SVM can map data that are not linearly classifiable in low-dimensional space to high-dimensional space by kernel functions. Then, the mapped data are classified and regressed. SVM possess strong generalization ability and can find a superior balance between complex non-linear mapping relations of limited data and generalization ability. The traditional support vector classification (SVC) is a typical binary classification model. The principle of the model is shown in Fig. 1. The mathematical theory can be given as^42^:

Suppose there are *n*-dimensional sample vectors in a region, then there is $w^{T} \cdot x + b = 0$ hyperplane, which divides the sample into two categories. The hyperplanes may exist in different forms and the one that satisfies the minimum distance between two types of samples is called the optimal hyperplane. The above condition can be given as Eq. (1):

1 $$[(w^{T} \cdot x_{i} ) + b]y_{i} \ge 1$$

where $w^{T}$ represents the weight vector, *b* denotes the bias of sample fitting deviation and $y_{i} \in \left\{ { - 1,\left. 1 \right\}} \right.$.

Figure 1 shows that the sum of two types of sample distances from the hyperplane is 2/||*w*|| and the hyperplane margin is equal to 2/||*w*||. Also, any training tuples that fall on hyperplanes H_1_ or H_2_, i.e., the sides defining the margin, are the support vectors, as shown in Fig. 1. Thus, the problem is the maximization of margin by minimizing the ||w||/2 value, which is a convex quadratic programming (QP) problem and can be solved with the help of Lagrangian operators.

2 $$L(w,b,\alpha ) = \frac{{\left\| w \right\|^{2} }}{2} - \sum\limits_{i = 1}^{k} {\alpha_{i} } [y_{i} (w^{T} \cdot x_{i} + b) - 1]$$

where: $\alpha_{i} > 0$ is Lagrange coefficient. In order to simplify the prediction calculation, the original problem is transformed into a mathematical dual problem by solving the partial differential of $w$, $b$ with the above formula.

3 $$MAX(\alpha ) = \sum\limits_{i = 1}^{k} {\alpha_{i} } - \frac{1}{2}\sum\limits_{i,j = 1}^{k} {\alpha_{i} \alpha_{j} } y_{i} y_{j} (x_{i} \cdot x_{j} )$$

The constraints of the above equation can be given as:

4 $$s.t.\left\{ \begin{gathered} \sum\nolimits_{i = 1}^{k} {y_{i} \alpha_{i} = 0} \hfill \\ \alpha_{i} \ge 0 \hfill \\ \end{gathered} \right.i = 1, \cdots ,k$$

When $\alpha_{i}^{*}$ is the optimal solution of the equation, then the optimal weight vector can be given as: $w^{*} = \sum\limits_{i = 1}^{k} {\alpha_{i}^{*} y_{i} x_{i} }$.

A unique solution under the given constraints exists when the problem satisfies $\alpha_{i} [y_{i} (w^{T} \cdot x_{i} + b) - 1] = 0$. The optimal classification plane function can be obtained by solving the question described as Eq. (5), where sgn() is a symbolic function.

5 $$f(x) = {\text{sgn}} \left[ {\sum\limits_{i = 1}^{k} {\alpha_{i}^{*} y_{i} (x \cdot x_{i} ) + b^{*} } } \right]$$

The relaxation variable $\xi_{i} \ge 0$ is introduced in the case of linear indistinguishability of the corresponding samples so that the maximum number of misclassified samples at the maximum classification interval can be satisfied under the given condition, as shown in Fig. 1. Then, Eq. (1) can be rewritten as:

6 $$[(w^{T} \cdot x_{i} ) + b]y_{i} \ge 1 - \xi_{i}$$

The penalty variable *C* is inserted in the constraint.

7 $$s.t.\left\{ \begin{gathered} \sum\nolimits_{i = 1}^{k} {y_{i} \alpha_{i} = 0} \hfill \\ C \ge \alpha_{i} \ge 0 \hfill \\ \end{gathered} \right.i = 1, \cdots ,k$$

For non-linear classification, the kernel function can map the sample data to $K(x_{i} ,x_{j} )$ high-dimensional space and, then, the problem of finding optimal hyperplane in the new space is simplified. Hence, the non-linear classification is realized. The Gaussian radial basis (RBF) is one of the mapping functions and, after selecting the kernel function, the problem can be given as Eq. (8):

8 $$MAX(\alpha ) = \sum\limits_{i = 1}^{k} {\alpha_{i} } - \frac{1}{2}\sum\limits_{i,j = 1}^{k} {\alpha_{i} \alpha_{j} } y_{i} y_{j} K(x_{i} \cdot x_{j} )$$

where $K(x_{i} ,x_{j} ){ = }\exp \left( { - g\left\| {x_{i} - x_{j} } \right\|^{2} } \right)$. The corresponding classification function can be given as Eq. (9):

9 $$f(x) = {\text{sgn}} \left[ {\sum\limits_{i = 1}^{k} {\alpha_{i}^{*} y_{i} K(x_{i} \cdot x) + b^{*} } } \right]$$

#### SVC multi-classifier

**Figure 2:**
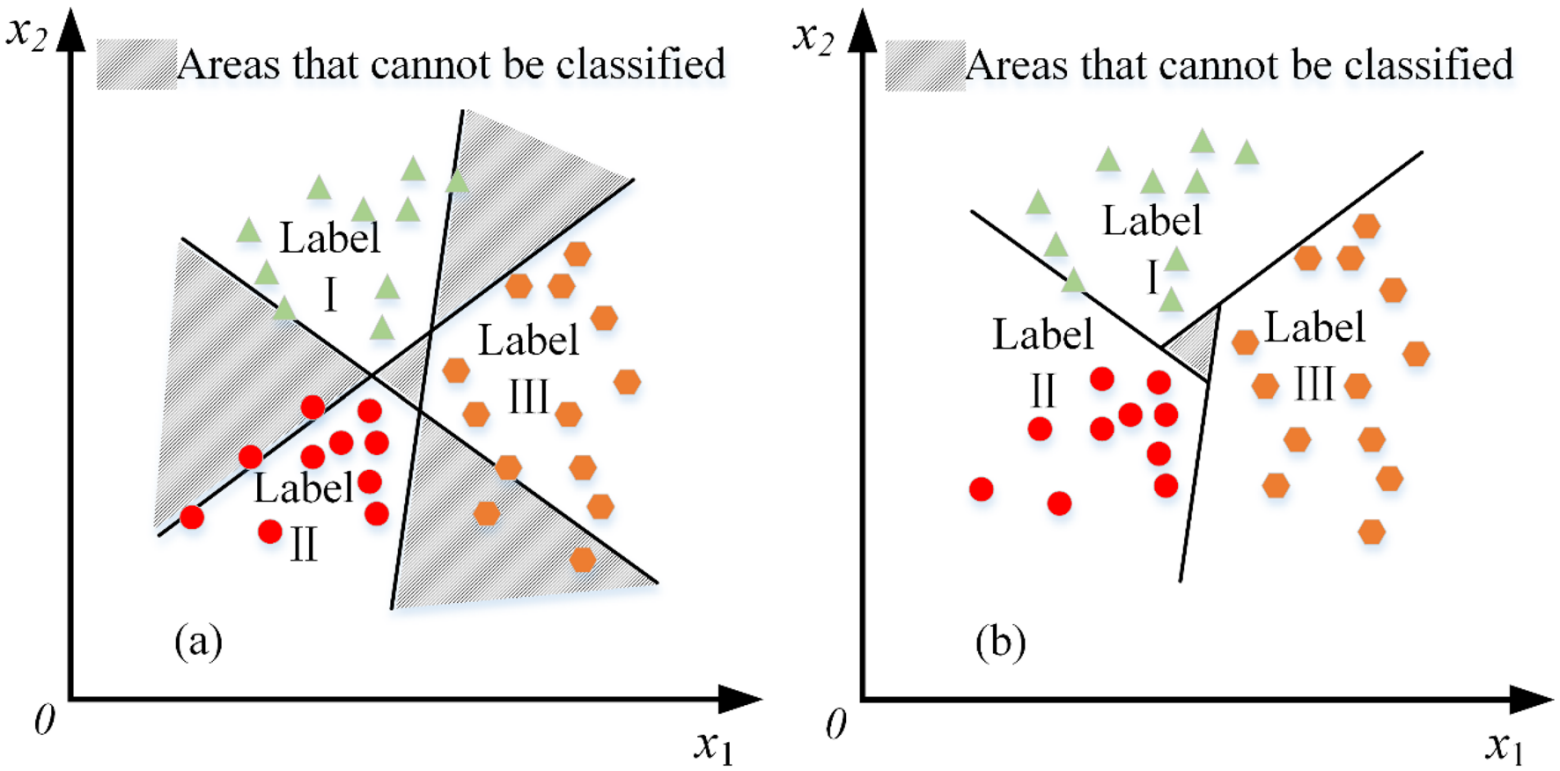
The schematic illustration of the SVC multi-classification algorithm (**a**, **b**).

In practice, the simple binary classification problems are less applicable and the rock mass classification is a typical multiclassification problem. Traditional SVC often utilizes a one-against-rest classification method in multiclassification problems. The basic principle is that each classification and remaining classifications form a binary calculation, and the *n* classifications build *n* sets of sub-classifiers. This multi-classification method utilizes the basic principle of SVC to achieve multi-classification, but the one-against-rest classification may bring too large non-distinguishable regions, resulting in an inferior generalization model, as shown in Fig. 2a. Therefore, the current study adopts the one-against-one classification method, as shown in Fig. 2b, which reduces the non-separable regions and enhances the generalization ability of the proposed model^43,44^.

### Heuristic optimization algorithm

In general, the SVM algorithm alone is inefficient. Hence, the optimized algorithms, such as Genetic Algorithm (GA) and Particle Swarm Optimisation Algorithm (PSO), were applied by some researchers to optimize the initial parameters of machine learning models and the increase in both predictive accuracy and convergence、speed of the constructed machine learning models after combining optimization algorithms has been demonstrated^45^. Grey wolf optimiser (GWO) is one of the latest heuristic algorithms. This new optimization method has shown a great result in optimizing problems and has successfully beaten the well-known methods such as the PSO in engineering design problems^46^. Therefore, this study uses three heuristic algorithms to optimize the SVM algorithm to explore the classification ability of different combinations of algorithms.

Optimization of SVC parameters using metaheuristic algorithms requires the construction of fitness functions to achieve optimal parameter selection. In SVM, classification accuracy (AR) and mean square error (MSE) are often used as fitness functions; AR or 1/AR is often used as a fitness function for classification models, and MSE is often used as a fitness function for prediction models^47^.

#### Genetic algorithms (GA)

At the University of Michigan, John and Bagley first proposed the genetic algorithm (GA), an optimization algorithm based on genetics and evolution theory^48^. GA is widely used in the field of optimization and optimal solutions. The core idea in GA is to utilize relevant information generated in the evolutionary history of a population to guide the search for results, leading to simplified application and excellent robustness^49^. The algorithm encodes the dataset, i.e., the population, and utilizes genetic operators to cyclically perform selection, crossover and mutation operations to generate new individuals, and constructs and calculates a reasonable fitness function for the selection of new populations to generate individuals, which satisfy the end conditions. Herein, the key parameters (*c*, *g*) in SVM are optimized using the genetic algorithm, as given below Table 2.

**Table 2:** Genetic algorithm.

Algorithm: genetic algorithm
(1) Data set processing
(2) Coding of initial population
(3) Computational fitness
(4) Population retention with excellent fitness
(5) Selection, crossover and mutation
(6) If the termination condition is satisfied, decoding is performed. If the termination condition is not satisfied, the algorithm returns to step 3
(7) Decode; output optimal solution (optimal *c*, optimal *g*)

The fitness function of the classification problem is:

10 $$F_{{{\text{fitness}}}} = \frac{{{\text{T}}_{n} }}{{\text{M}}}\,(n = {\text{I}},{\text{II}},{\text{III}},{\text{IV}},{\text{V}})$$

where T_*n*_ = The number of accurate classifications in the training and test sets; and M = Total number of samples in the training and test sets.

#### Particle swarm optimization (PSO)

Eberhart and Kennedy proposed the principle of bird flocks foraging in 1995^50^. This theory evolved into an optimization algorithm for group intelligence. PSO is often used in the optimization of algorithms through cooperation and competition between particles in a population. PSO renders efficient parallel search capabilities, tracks in real-time, and adjusts the search methods in real-time. PSO optimizes the SVM in a similar way to GA using the following algorithmic steps, as given below Table 3. The PSO algorithm optimizes the fitness function of the SVC algorithm in the same way as Eq. (10).

**Table 3:** Particle swarm optimization.

Algorithm: particle swarm optimization
(1) Data set processing
(2) Determination of fitness function
(3) Particle initialization and PSO parameters setting
(4) Computing the fitness function value of each particle
(5) If the termination condition is satisfied, output is the optimal solution. If the termination condition is not satisfied, step 6 is executed
(6) Speed update; individual update
(7) If the termination condition is satisfied, output is the optimal solution. If the termination condition is not satisfied, step 6 is re-executed
(8) Output optimal solution (optimal *c*, optimal *g*)

#### Grey wolf optimizer (GWO)

The gray wolf algorithm was proposed by Mirjalili et al. in^51^. The algorithm mimics wolf hunting hierarchy and works on simple computational principles and few control parameters. Mirjalili et al. have compared GWO with PSO, GSA, DE, EP and ES based on 29 well-known test functions, showing the promise of GWO^51^. The optimization process is given below Table 4. The fitness function of the classification problem is^47^:

11 $$F_{{{\text{fitness}}}} = \frac{1}{{{\text{AR}}_{tr} }} + \frac{1}{{{\text{AR}}_{va} }}$$

where AR_*tr*_ = classification accuracy of training sets in the training process; and AR_*va*_ = classification accuracy of validating sets.

**Table 4:** Grey wolf optimizer.

Algorithm: grey wolf optimizer
(1) Initialize search space, set the number of wolves *N*, set the maximum number of iterations, random initialization (*c*, *g*).
(2) Traverse the gray wolf population, calculate the degree of individual adaptation, establish social order of population according to the degree of adaptation, and classify the gray wolves with higher degrees of adaptation into α-wolf, β-wolf, δ-wolf, and the remaining into ω-wolf.
(3) Calculate the spatial distance of each ω-wolf from α, β and δ wolves and update the spatial position of α, β, and δ wolves and the corresponding prey.
(4) If the termination condition is satisfied, output is the optimal solution; if not, return to the third step to update the position.
(5) Output optimal solution (optimal *c*, optimal *g*)

## Database description and SVC-based model development

### Rock mass quality characterization factors

The selection of input parameters is the first step in building an assessment model for rock mass quality using the AI models. Santos et al. applied a technique of multivariate statistics, the factor analysis, to select a set of variables commonly used in rock mass classifications. The most important variables for describing the rock mass quality were kept in the model, and the removal of less important variables was justified. Studies have demonstrated that several factors affect the rock mass quality, such as rock strength, integrity of the rock mass and rock environment (including the groundwater seepage)^52^. Therefore, firstly, in this paper, we refer to the three major variables in the literature and select rock saturated compressive strength (R_c_), etc. as rock strength characterization parameters, RQD value and rock mass integrity factor (K_v_) as rock discontinuities' characteristics, unit roadway water inflow (ω/(L(min 10 m)^−1^)) as the characterization parameter of groundwater. The input parameters should reflect the characteristics of rock strength, degree of rock fragmentation, degree of structural surface development of rock body, and weakening effect of groundwater on rock body. Meanwhile, the above four parameters are used as the input parameters of the model.

### Database creation and analysis

Artificial intelligence can establish non-linear relationships between multiple factors by learning from a large dataset. Therefore, the samples used as a training set must be representative of all the categories. Currently, many case data for rock mass classification are documented in the literature. Thus, mining the results of previous studies can yield a large number of valid samples. Furthermore, the model accuracy can be improved by expanding the sample size.

Herein, we investigate and analyze the relevant work throughout the past years. The available data were filtered to obtain 80 sets of tagged data, which were assembled into the database for SVC model training (Table 5). All data were obtained from the available literature. It contains rock samples from different geographical areas and engineering types in China. For example, the sample sources include power station underground works, tunnel envelope, roadway envelope, etc. The established database satisfies the model training requirements. The samples are divided into two parts, where 64 groups are randomly selected as a training set and the remaining 16 groups are set as a testing set. The better training samples were determined after several random training sessions. The markers of the training and prediction samples are shown in Table 5.

**Table 5:** Basic data for rock mass classification of some underground projects around the world.

No.	Cases	R_c_/(MPa)	RQD%	K_v_	ω/[L (min 10 m)^−1^]	Actual grade	References	Remarks
1.	Surrounding rock of underground engineering area of Guangzhou Pumped Storage Power Station^15^	90.1	71.8	0.57	0	II	Zhou et al. (2016)^15^	Training sample
2.		40.2	51	0.38	10.5	III		Training sample
3.		25	52	0.22	12	III		Training sample
4.		90	68	0.38	21	III		Test sample
5.		45	51	0.15	5	III		Training sample
6.		95	76	0.7	12	II		Training sample
7.		95	87	0.7	9.8	II		Training sample
8.		90	76	0.57	11	II		Test sample
9.		70.5	35	0.35	10	III		Training sample
10.		35	50	0.3	20	III		Training sample
11.		90	68	0.57	18.5	III		Training sample
12.		95	82	0.7	0	II		Training sample
13.		87.3	75	0.3	0	II		Training sample
14.		70.5	52.5	0.6	15	III		Test sample
15.		8.4	30.2	0.18	50	V		Training sample
16.		36	26	0.22	5	IV		Training sample
17.		40.2	50	0.5	10	III		Test sample
18.		90	71	0.35	18	III		Training sample
19.		95	75	0.7	0	II		Test sample
20.		90	77.5	0.57	10	II		Training sample
21.		20	31.5	0.23	46	IV		Training sample
22.		34	50.9	0.32	21	III		Training sample
23.		90	75.5	0.45	8	II		Test sample
24.		95	80	0.5	0	II		Training sample
25.		92	78.5	0.55	6	II		Training sample
26.		93	85	0.6	0	II		Training sample
27.		70	30.2	0.4	10	III		Training sample
28.		95	87	0.5	0	II		Training sample
29.		96	82	0.75	0	II		Test sample
30.	Surrounding rock of tunnel in underground engineering area^16^	130.5	78	0.75	10	III	Hu et al. (2012)^16^	Training sample
31.		28.6	52.5	0.38	23	IV		Training sample
32.		200	100	1	0	I		Training sample
33.		180	97.5	0.94	1.3	I		Training sample
34.		160	95	0.88	2.5	I		Test sample
35.		105	86.3	0.68	6.3	II		Training sample
36.		75	78.8	0.53	8.8	II		Training sample
37.		60	75	0.45	7.5	III		Training sample
38.		52.5	68.8	0.41	13.8	III		Training sample
39.		37.5	56.3	0.34	21.3	III		Training sample
40.		26.3	43.8	0.28	50.6	IV		Test sample
41.		18.8	31.3	0.23	100	IV		Training sample
42.		11.3	18.8	0.15	169	V		Training sample
43.		7.5	12.5	0.1	213	V		Test sample
44.		0.8	6.3	0.05	256	V		Training sample
45.		70	50	0.5	5	III		Test sample
46.		34	50.9	0.32	21	III		Training sample
47.	Rock mass engineering of underground stope in Sijiaying Iron Mine^53^	181.73	58.13	0.47	17	II	Hu et al. (2017)^53^	Training sample
48.		101.73	34.97	0.54	109	III		Training sample
49.		98.35	32.28	0.51	18	III		Training sample
50.		82.17	39.93	0.53	168	IV		Training sample
51.		105.23	53.3	0.37	223	III		Training sample
52.	No. 2 diversion tunnel at left abutment of Manwan Hydropower Station^54^	140.0	92.5	0.81	3.8	I	Yang et al. (1999)^54^	Training sample
53.		120	90	0.75	5	II		Training sample
54.		90.0	82.5	0.60	7.5	II		Training sample
55.		45.0	62.5	0.38	17.5	III		Training sample
56.		37.5	56.3	0.34	21.3	III		Training sample
57.		30	50	0.30	25	III		Test sample
58.		26.3	43.8	0.28	50.0	IV		Training sample
59.		22.5	37.5	0.25	75.0	IV		Training sample
60.		15	25	0.20	125	IV		Training sample
61.		3.8	6.3	0.05	256.3	V		Training sample
62.		40	25	0.22	20	IV		Training sample
63.		72	90	0.57	10	II		Training sample
64.		51	40	0.38	10	III		Training sample
65.		28	40	0.32	20	IV		Training sample
66.		51	25	0.15	20	IV		Training sample
67.	An underground project in Liaoning^55^	185.5	0.12	0.89	6	II	Lijian et al. (2014)^55^	Training sample
68.		176.4	0.27	0.8	8	II		Test sample
69.		158.2	0.08	0.94	6	II		Training sample
70.		201.1	0.04	0.97	5	I		Training sample
71.		181.9	0.24	0.92	9	II		Training sample
72.	Deep rock mass of Sanshandao Gold Mine^56^	95	79.8	0.65	5	III	Liu et al. (2011)^56^	Training sample
73.		95	88.6	0.4	70	III		Training sample
74.		95	85.6	0.65	25	III		Test sample
75.		118	90.1	0.7	10	II		Training sample
76.		118	89.5	0.55	45	IV		Training sample
77.		80	82.5	0.5	35	IV		Training sample
78.	Pingzitou tunnel rock mass^57^	68	75.4	0.55	30	III	Huang et al. (2012)^57^	Training sample
79.		50	55.6	0.4	20	IV		Training sample
80.		15	16	0.2	125	V		Training sample

Most of the rock mass grades in Table 5 are based on traditional rock mass classification methods. The different rock mass quality is qualitatively described as the following Table 6^1^.

**Table 6:** Classification reference table.

Rock mass quality grades	Qualitative description of rock quality
I	Extremely hard rock and intact rock masses
II	Extremely hard or hard rock and intact rock masses Relatively hard rock and intact rock masses
III	Extremely hard or hard rocks and relatively broken rock masses Relatively hard or soft-hard rock and relatively intact rock mass Relatively soft rocks and intact rock masses
IV	Extremely hard or hard rock and broken rock Extremely hard or hard rock and broken rock Relatively soft rocks and relatively broken or intact rock masses
V	Soft rock and intact or relatively intact rock masses Relatively soft rocks and fractured rock masses Soft rock and relatively broken or fractured rock masses Extremely soft rock and extremely fractured rock masses

**Figure 3:**
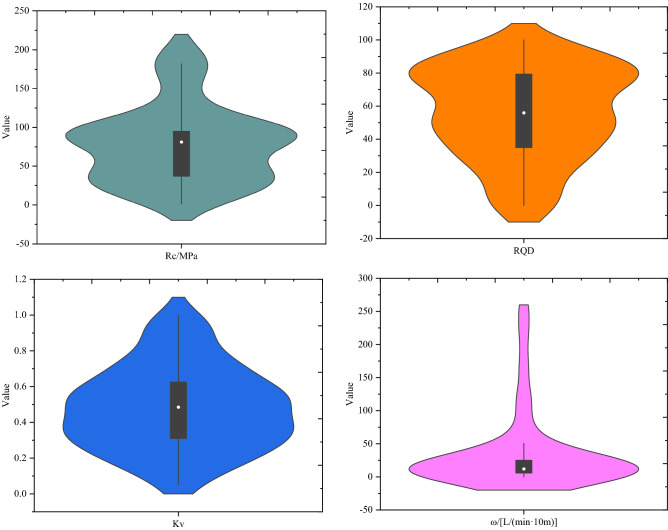
The database violin diagram.

**Figure 4:**
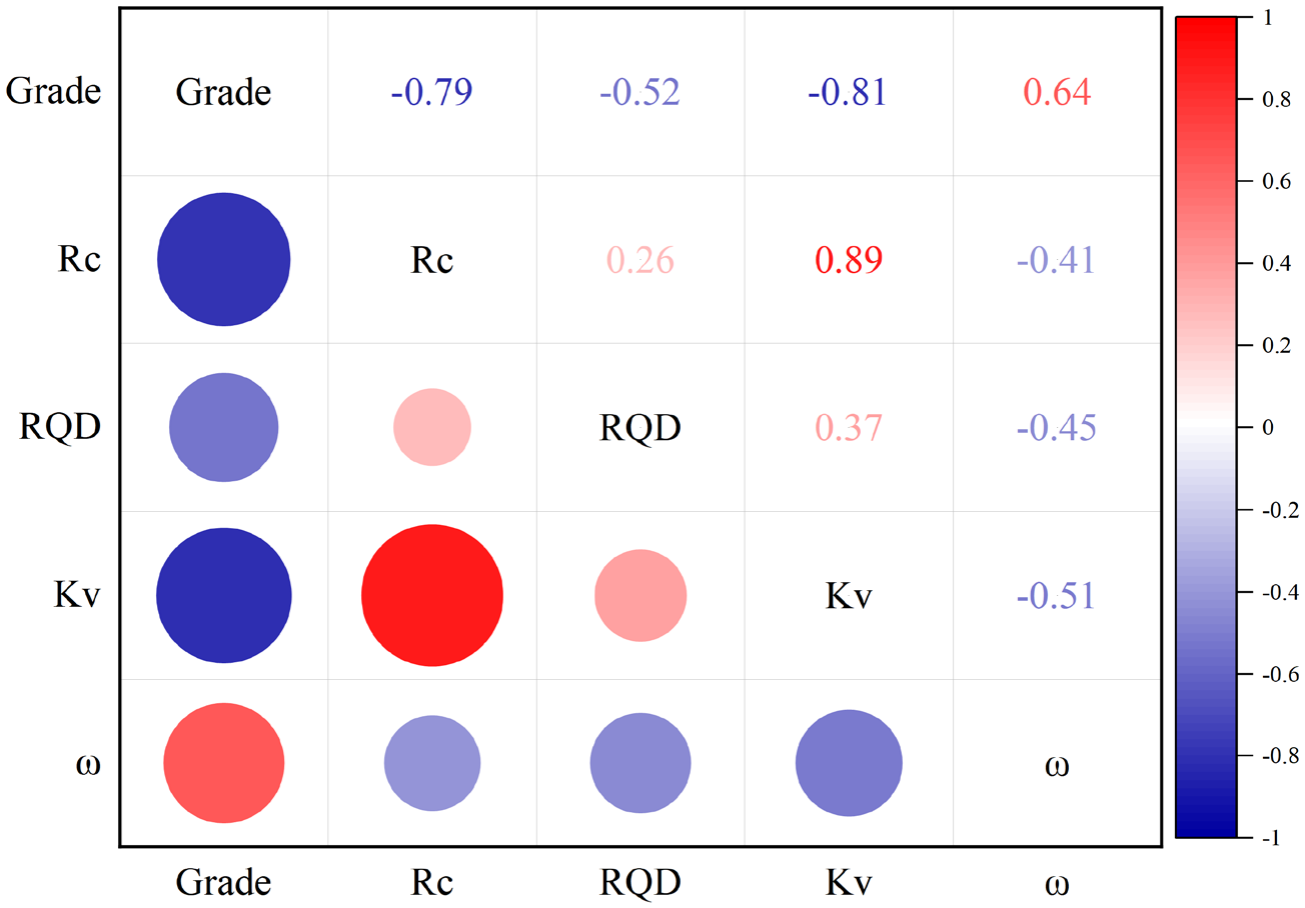
The database correlation matrix.

Then, the numerical analysis was performed on input parameters in the database and the analysis results are shown in Table 7. The single-factor analysis results of each input parameter are shown in Fig. 3. Figure 3 contains line box plots and violin plots for each factor, where the line box plots reflect the data interval, interquartile range and median data for each indicator, and the violin plots highlight the distribution of dataset. As shown in Fig. 3, the median value of each indicator is not in the center of the data box, which indicates the asymmetrical data distribution. At the same time, we conducted a correlation analysis of the factors in the dataset, and the correlation matrix is shown in Fig. 4. Figure 4 shows that the p-values of each factor and rock mass quality grade are all greater than 0.5. Among them, R_c_, RQD, and K_v_ show a negative correlation trend with rock mass quality grade, indicating that the larger the three parameters are the lower the grade is, the better the rock mass quality is. On the contrary, ω showed a positive correlation. In general, the selected factors showed a good correlation with rock mass quality. Moreover, the density deviation of ω distribution is large, which indicates the specificity of sample data. In general, the sample set constructed in this paper meets the model training requirements.

**Table 7:** Descriptive statistics of input parameters with the range, mean, standard deviation and skew for SVC modeling.

Parameter	Mean	Median	Min	Max	Standard deviation
R_c_	77.99	81.09	0.8	201.1	49.67
RQD	55.95	55.95	0.04	100	27.81
K_v_	0.48	0.485	0.05	1	0.23
ω/[L (min 10 m)^−1^]	35.48	12	0	256.30	58.47

### SVC-based model development

In underground projects, such as mining and tunneling, the safety of production and control of construction cost are highly dependent on the quality of rock mass^58^. Therefore, we must classify the rock mass according to the project needs. To ensure a smooth project, different rock mass quality classes correspond to different construction methods and support measures. Several factors reflect the quality of rock masses in actual projects. Therefore, the researchers refer to different grading criteria and factors when building the rock mass quality assessment models, leading to a poor generalization of the results. Moreover, the continuous work on new models resulted in a lot of duplication research and bad utilization of the existing data.

**Figure 5:**
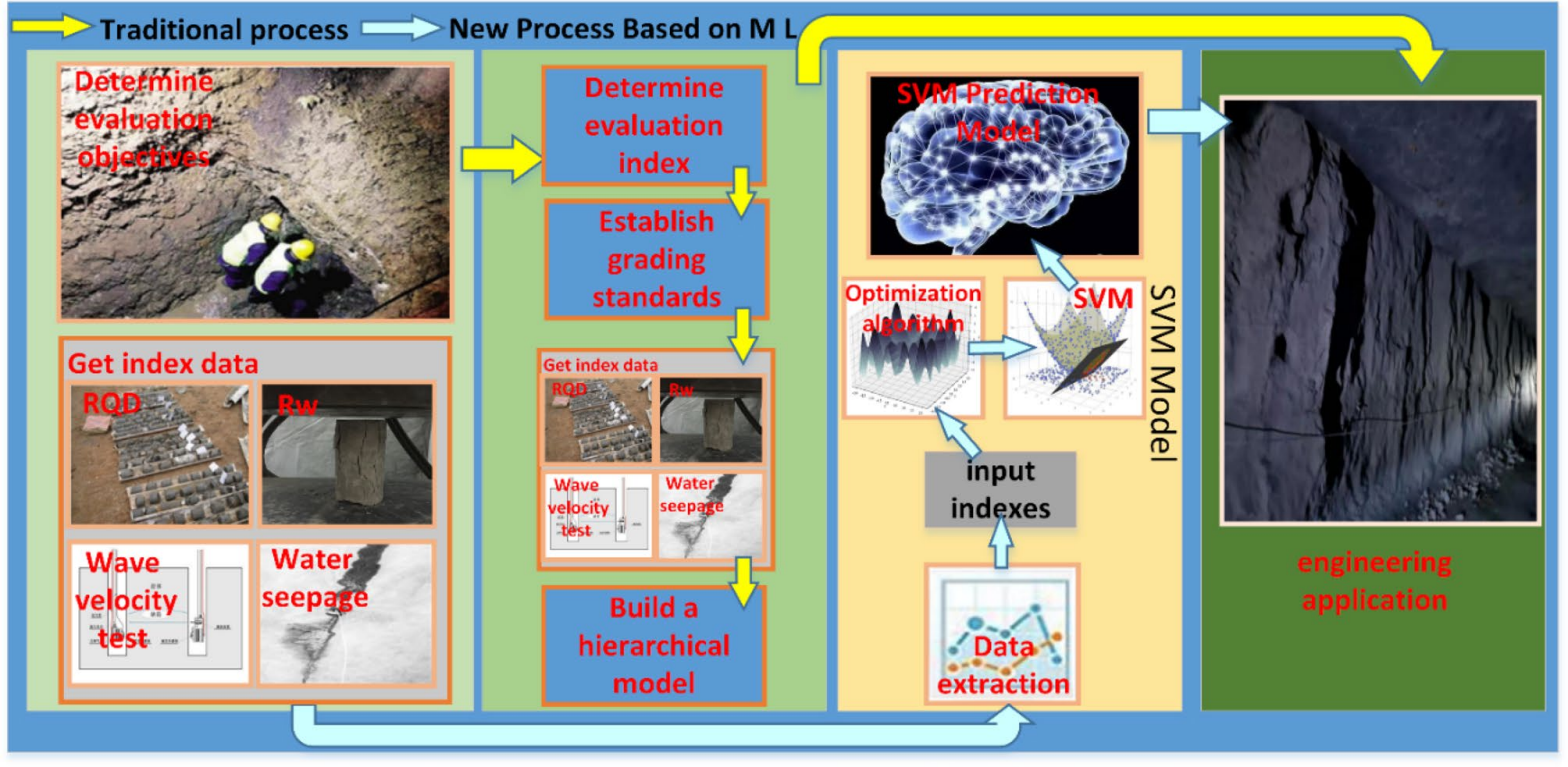
The prediction case flow chart.

Therefore, the current study establishes the SVC classification model of rock mass by a large number of prior cases. We expect to replace the complex and repetitive modeling, and classification work by optimized SVC with artificial intelligence algorithms in engineering practice. It is worth emphasizing that the prediction results will become more accurate with the continuous use of the proposed SVC model. The implementation processes of the traditional rock mass classification model and AI classification model are compared in Fig. 5. The process represented by the yellow arrows in Fig. 5 is the steps of rock mass classification using conventional methods. First, the rock mass quality characterization factors are determined by the actual conditions. Secondly, obtain the grade interval of each factor. Third, the values of each factor are obtained. Fourth, obtain the rock mass quality grade by a classification model. The process represented by the blue arrow in Fig. 5 is the step of rock mass classification using SVC methods. When using the SVC model, the rock mass quality grade is obtained by simply inputting the values of the characterization parameters into the trained model. It is worth emphasizing that the complex process needs to be repeated each time the traditional method is used. In contrast, it is much easier to use SVC classification. As the model continues to be used, its accuracy and generalization capabilities are continuously improved.

**Figure 6:**
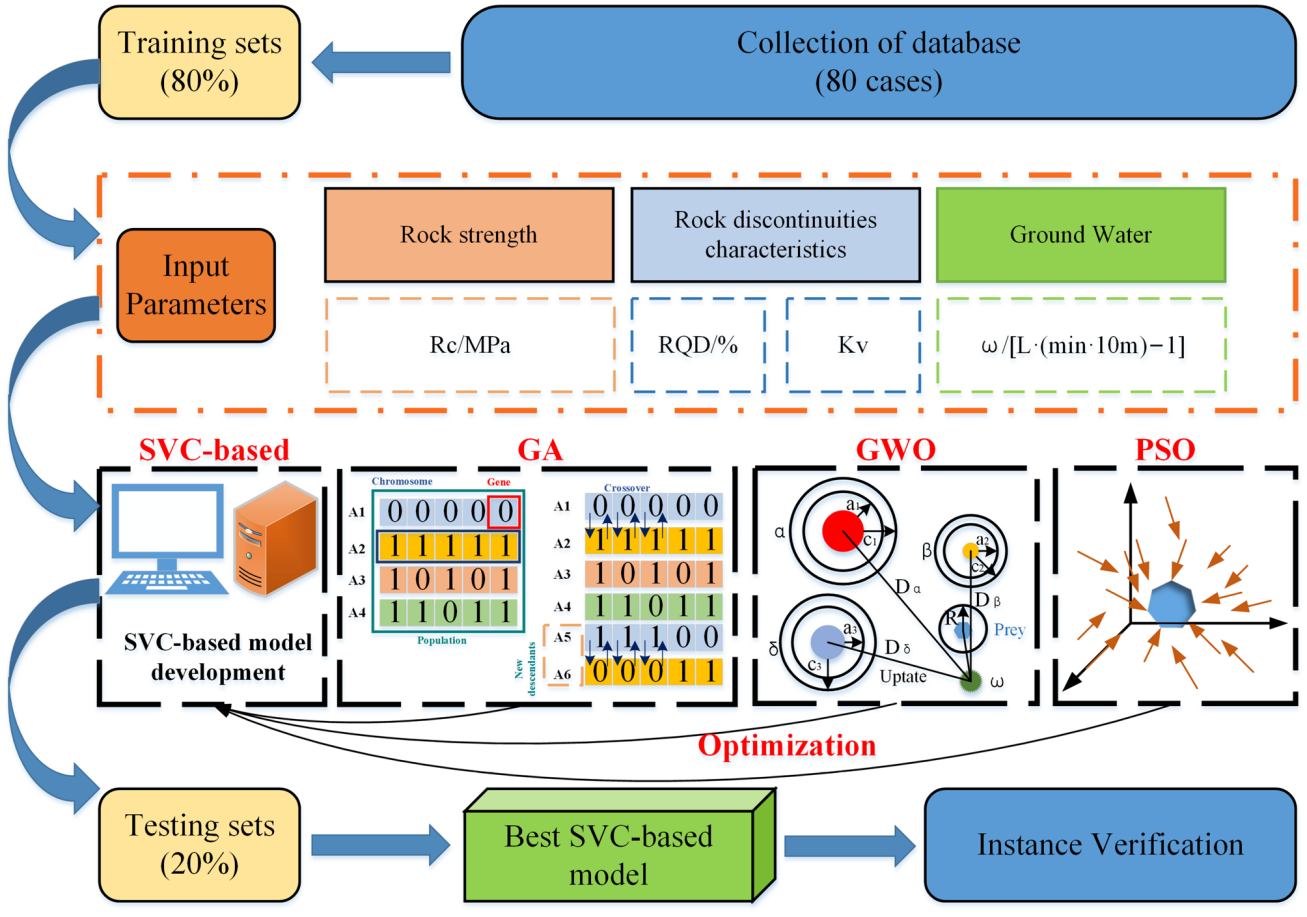
The research architecture for the proposed SVM-based approach with GWO, GA and PSO optimization method.

The optimized SVC model for rock mass classification is established based on SVC theory and database. The model is built and trained using the MATLAB software and the main part is based on the SVM algorithm using the LIBSVM toolbox^59,60^. Herein, the heuristic algorithms, such as GA, PSO and GWO, are utilized to optimize SVC, reduce prediction error, and improve computing efficiency and generalization ability. By comparing the classification performance of three algorithms, the heuristic algorithm with SVC, which renders a better optimization effect, is selected to construct the rock mass classification model. The model training input metrics are the rock mass quality characterization parameters, as identified in “Rock mass quality characterization factors”. The output index is the rock mass quality grade. The optimized SVC model comparison and construction process are shown in Fig. 6.

**Figure 7:**
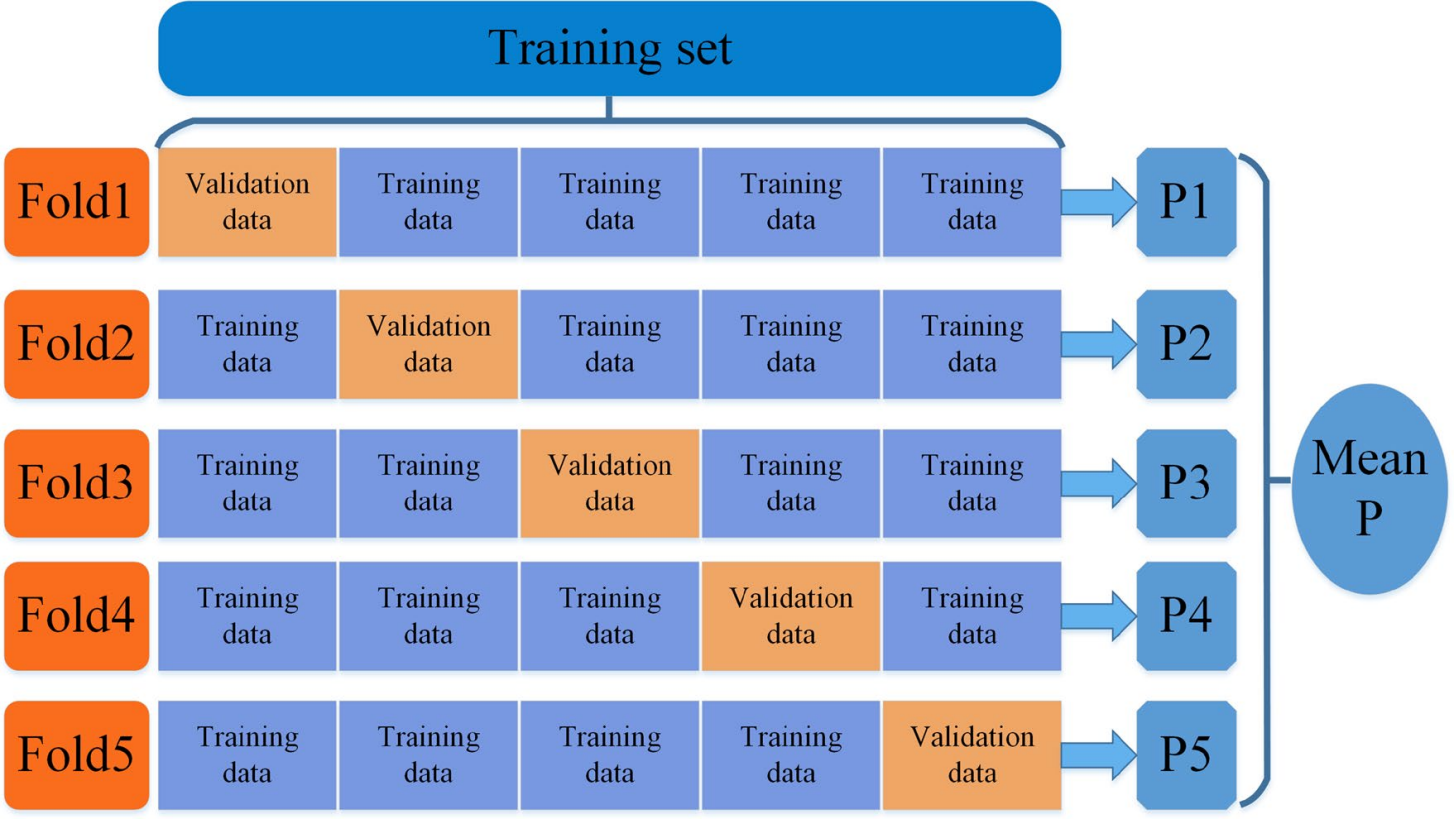
A schematic diagram of fivefold cross-validation.

Cross-validation is a useful method for assessing the model robustness and generalization. It can avoid overfitting the model when the samples are small. In this study, we use the fivefold cross-validation, where we divide the training set into five samples, one for training and four for testing. This process is operated five times, and the average classification accuracy is the model accuracy. A general display of fivefold cross-validation is shown in Fig. 7. In this figure, P1, P2, P3, P4 and P5 represent the prediction results of the corresponding fold, respectively.

## Results and discussion

### SVC- based model classification performance

For the multi-classification problem, two main parameters affect the SVC classification effect, i.e., the error penalty parameter (*c*) and the kernel function parameter (*g*). The role of penalty parameter *c* is to adjust the learning confidence range and empirical risk ratio in a defined data subspace, rendering better generalization. The optimal *c* differs in different data subspaces. The kernel function parameter *g* mainly influences the complexity of the degree of distribution of sample data in high-dimensional space. The three models are trained and tested according to the database and the optimization ability of three heuristics is evaluated based on the test results. The performance classification metrics in the multi-classification model, such as accuracy, precision, recall, F_1_ value and model operation time consumption, are used to evaluate the model. Among them, accuracy is a direct response to the model prediction performance. The model computation time reflects the ease of model running. The rest of the metrics reflect the classification ability of the model itself. The initial process of self-organization-based heuristic optimization-seeking algorithm is stochastic, which means that the combinatorial model first obtains a certain key parameter value and search it within the fitness criterion to obtain the optimal parameter. The combined rock mass classification model was subjected to the training parameter settings.

1. GA-SVC: Set the maximum number of iterations of the genetic algorithm to 100, set the maximum number of populations to 20, and set both *c* and *g* to [0, 100] for the merit search range. The mutations are performed to alter the binary code from 0 to 1 or vice versa. Hence, the rate of mutation is set at 0.05 and the crossover probability is 0.9.
2. PSO-SVC: Set the number of search groups to 20 and the maximum number of iterations to 100; set the search range of both *c* and *g* to [1, 100]; and set the personal factor c_1_ to 1.5 and the social factor c_2_ to 1.5.
3. GWO-SVC: The number of wolf packs is set to 20, the maximum number of iterations is 100, and the range of key parameter penalty coefficient *c* and the kernel parameter coefficient *g* are both searched in the range of [1, 100].

The current study normalizes the training data to eliminate the effect of dimension. The parametric search is conducted using the processed data and the model is validated using a fivefold-cross-validation of training samples to determine the SVC key parameters *c* and *g*. According to the previous studies, the choice of RBF kernel function can lead to better model training results. One should note that different kernel function choices are available in the LIBSVM tool.

**Figure 8:**
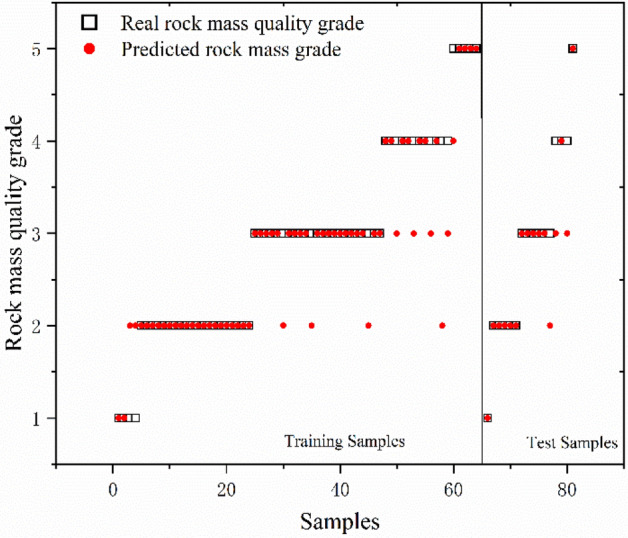
GA-SVC: predicted sample *vs.* actual sample.

**Figure 9:**
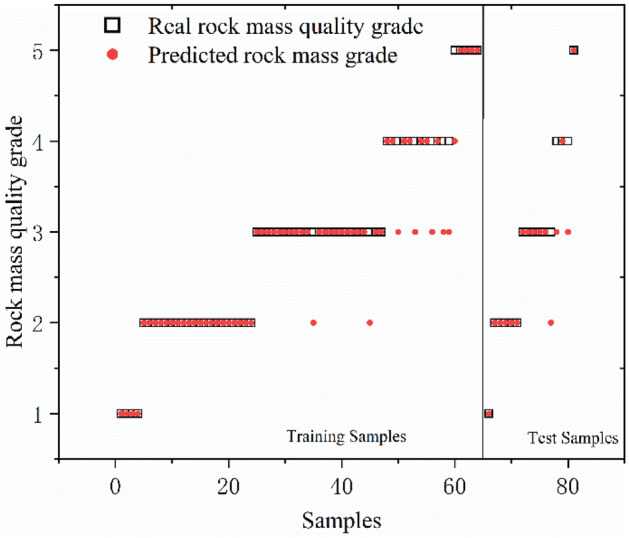
PSO-SVC: predicted sample *vs*. actual sample.

**Figure 10:**
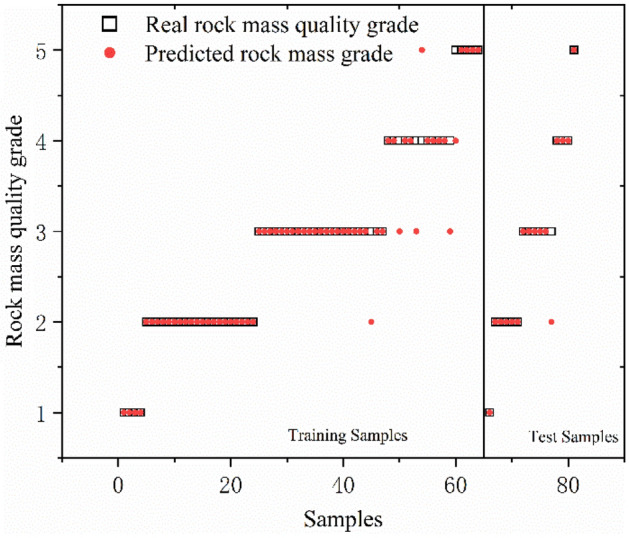
GWO-SVC: predicted sample vs. actual sample.

**Figure 11:**
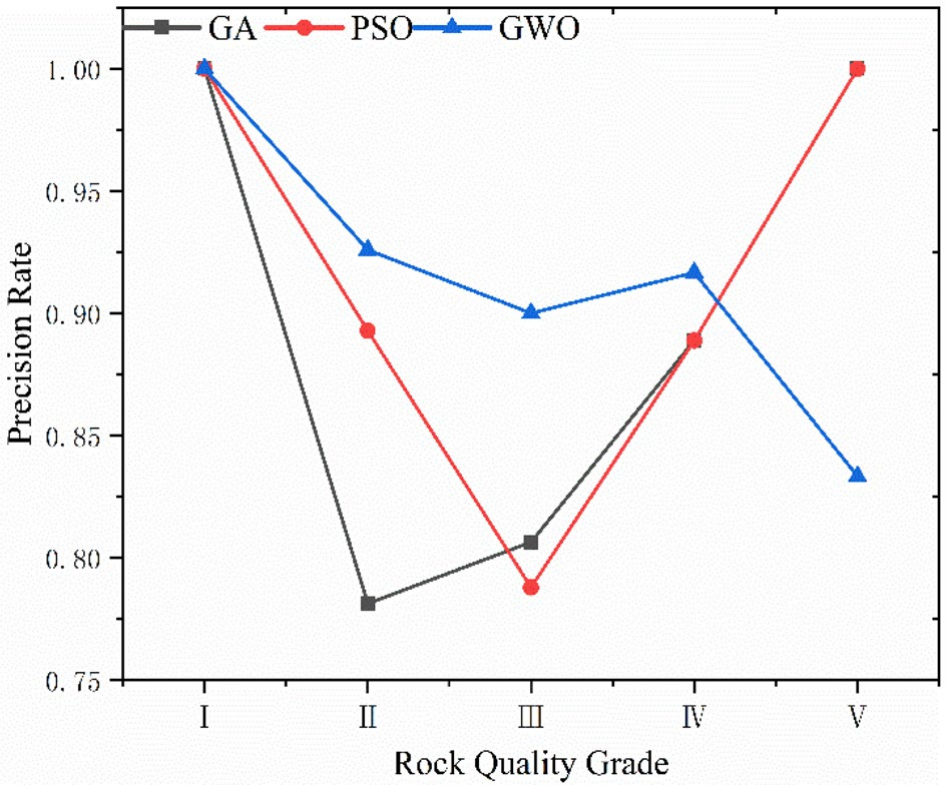
Classification precision.

**Figure 12:**
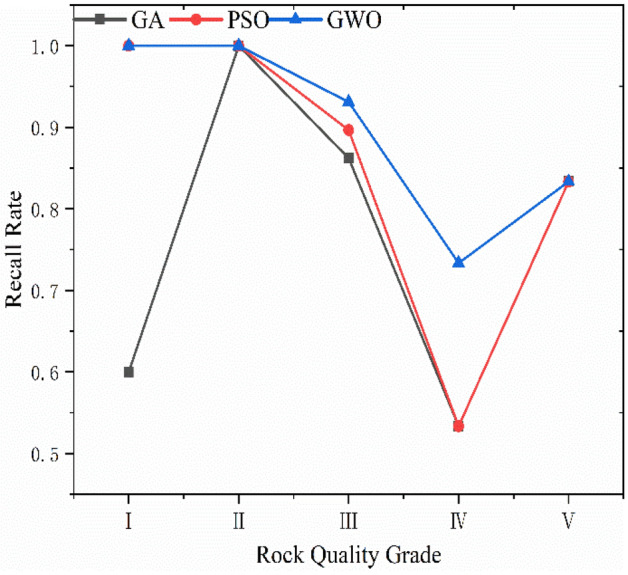
Classification recall.

**Figure 13:**
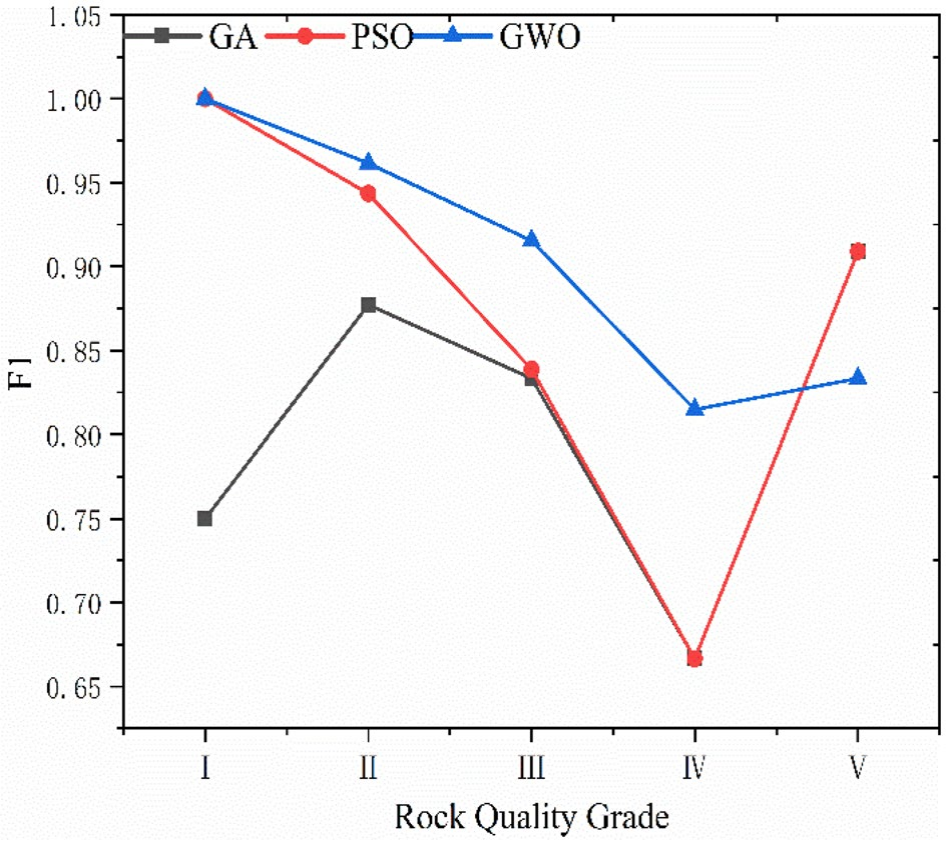
Classification F_1_ value.

GA, PSO and GWO optimization selected the key parameters of SVC. The results reveal that the genetic algorithm determines Optimal *c* = 4.734 and Optimal *g* = 3.7127; the particle swarm algorithm determines Optimal *c* = 62 and Optimal *g* = 0.5501, and the gray wolf algorithm determines Optimal *c* = 22.1397 and Optimal *g* = 2.8339. The SVC models were trained using the resulting optimal parameters and the results of three combined models for database classification are shown in Figs. 8, 9, and 10 and Table 8. The box in the figure represents the real rock mass quality grade, the red-colored dot represents the model prediction grade, the left side of the black-colored vertical line presents the prediction result of the training set, and the right side shows the prediction result of the test set. The results demonstrate that the three heuristic algorithms possess different abilities to optimize SVC. In terms of training set validation accuracy, all three combined models are more than 80% accurate and render superior performance. Among them, the GWO-SVC algorithm results in optimal performance with 90.6250% (58/64) accuracy of the training set prediction, followed by PSO-SVC with 87.5000% (54/64) accuracy of the training set prediction and GA-SVC with 82.8125% (53/64) accuracy of the training set prediction. This shows that all three models can achieve the rock mass classification function with reasonable accuracy and reliability through training. The training results of GWO-SVC algorithm with the current training set outperform the other two optimization-seeking algorithms. The trained model was used to test the classification of 16 datasets and the classification results are presented in the right-hand panels (Figs. 8, 9, 10). The GWO-SVC rendered the highest model classification accuracy (93.7500%, 15/16), whereas both PSO-SVC and GA-SVC exhibited similar performance, i.e., 81.2500% (13/16) and 81.2500% (13/16), respectively. The rock mass classification is such a multi-classification problem that the model's ability to classify different categories should be assessed while considering the accuracy. Herein, the precision, recall and F_1_ values are calculated for different grades (I–V) of the three models. By analyzing and comparing the classification capabilities of different grades of the model, it is possible to determine that which type of rock mass level lacks training at this stage, providing guidance for further model optimization. Figures 11, 12 and 13 show the discrimination accuracy, recall, and F_1_ values for each category of three combined models, respectively. Figure 11 and 12 show that the three combined algorithms differ in their ability to classify different grades. The classification accuracy rate of all three optimization algorithms for Grade I rock samples is 1. The recall rate of PSO-SVC and GWO-SVC is 1, whereas the recall of GA-SVC is 0.6. At this stage, it indicates that the PSO-SVC and GWO-SVC judge all Grade I samples correctly and, instead of misclassification, the GA-SVC exhibits under-classification. Under-classification means that all samples of Grade I are not found. The classification precision of all three optimization algorithms for Grade I rock samples is 1. The recall of PSO-SVC and GWO-SVC is 1, while the recall of GA-SVC is 0.6. At this point, it means that PSO-SVC and GWO-SVC judge all the samples of Grade I correctly, and GA-SVC does not misclassification, but there is under- classification. Therefore, the order of classification ability of Grade I samples is PSO-SVC = GWO-SVC > GA-SVC. For the Grade II rock samples, all three algorithms correctly identified the real Grade II rock samples. However, there are different degrees of misclassification. Misclassification means that samples of other grades are divided into Grade II. Therefore, the order of classification ability of Grade II samples is GWO-SVC > PSO-SVC > GA-SVC. For the Grade III rock samples, all three algorithms suffer from both under-classification and misclassification. From the precision and recall viewpoints, Grade III classification ability can be given as GWO-SVC > PSO-SVC > GA-SVC. The precision and recall of PSO-SVC and GA-SVC in Grade IV were the same, i.e., 0.89 and 0.53, and GWO-SVC were 0.92 and 0.73, respectively. Therefore, the order of classification ability of Grade IV samples is GWO-SVC > PSO-SVC = GA-SVC. The precision and recall rates of PSO-SVC and GA-SVC for Grade V samples are the same, i.e., 1 and 0.83, respectively, confirming that both algorithms do not exhibit misclassification. However, under-classification of Grade V samples still exists. The precision and recall of GWO-SVC were both 0.83, indicating partial under-classification and misclassification. Therefore, the order of classification ability of Grade V samples is GWO-SVC > PSO-SVC = GA-SVC. Combined with the F_1_ values in Fig. 13, the F_1_ values of Grades I, II, III and V of the three algorithms are better than the Grade IV, indicating the inferior classification ability of the given algorithms for Grade IV samples. The poor quality of Grade IV samples cannot sufficiently train the model and results in inferior classification ability. The F_1_ value of PSO-SVC and GWO-SVC for Grade I samples is found to be 1, indicating the absence of misclassification and under-classification. In summary, the classification ability of three algorithms on different grades can be ranked as: GWO-SVC > PSO-SVC > GA-SVC.

**Table 8:** The variables and summary of as-proposed models for SVC.

Algorithm	Best *c*	Best g	Sample	Accuracy %	T (s)
GA-SVC	4.734	3.7127	Train set	82.8125% (53/64)	6.03
			Test set	81.2500% (13/16)	
PSO-SVC	62	0.5501	Train set	87.5000% (54/64)	4.30
			Test set	81.2500% (13/16)	
GWO-SVC	22.1397	2.8339	Train set	90.6250% (58/64)	1.54
			Test set	93.7500% (15/16)

The operation speed reflects the algorithm's optimization ability and a faster operation speed renders optimal performance in training large-sized samples. Herein, the computational speed of GA-SVC, PSO-SVC and GWO-SVC for 100 iterations was found to be 6.03 s, 4.30 s and 1.54 s, respectively. These results reveal that the GWO-SVC model has an advantage in training and prediction time consumption. We have analyzed the accuracy, precision, recall, F_1_ value and computational time consumption of the three models in detail. Overall, the GWO-SVC rendered the best classification performance, followed by PSO-SVC and GA-SVC.

### Sensitivity analysis

For exploring and comparing the sensitivity of diferent infuenced factors on rock mass quality, in this section, the cosine amplitude method was employed^38^ . Each input variable and one output variable were transformed into a single column matrix. Thus, five single column matrixes were obtained as Eq. (12).

12 $$x_{a} = \left\{ {x_{a1} ,x_{a1} ,...,x_{an} } \right\}$$

where the length of each single column matrix is equal to the number of all datasets and then the sensitivity of diferent infuenced factors on rock mass quality can be calculated as Eq. (13)

13 $$s_{ab} = \frac{{\sum\nolimits_{n = 1}^{80} {x_{an} x_{bn} } }}{{\sqrt {\sum\nolimits_{n = 1}^{80} {x_{an}^{2} } } \times \sqrt {\sum\nolimits_{n = 1}^{80} {x_{bn}^{2} } } }}$$

**Figure 14:**
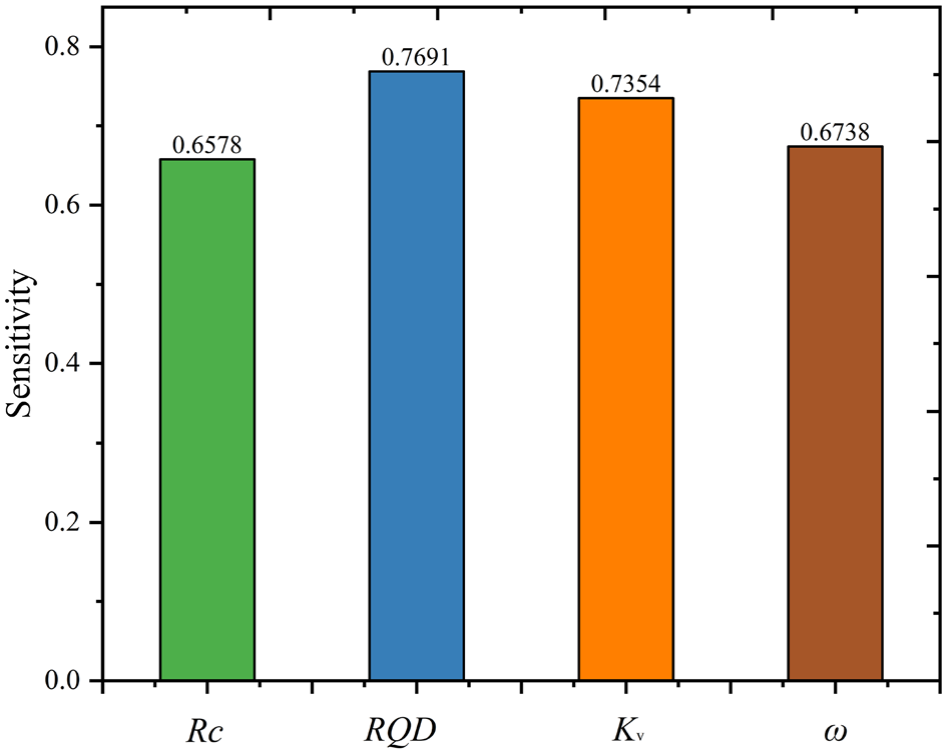
Sensitivity analysis of different factors on rock mass quality.

According to the results (Fig. 14), it can be observed that the most sensitive factor is RQD and Kv, the RQD is more important. This result is reasonable because the degree of rock fragmentation also plays a large part in the traditional classification method. Finally, the sensitivity of diferent parameters on rock mass quality can be sorted in descending order as: RQD, K_v_, ω, R_c_.

## Model verification

### Validation case: chambishi copper mine

Chambishi copper mine is a major mining project of the China Nonferrous Metals Group in Zambia. The copper mine is located in the central part of the Zambian copper belt, which is on the northern edge of the Chambishi Basin. The currently developed artificial intelligence model is mainly applied to the southeast orebody of the Chambishi copper mine. The southeast ore body is located about 7 km to the southeast of the main mining area, which is 6 km long (from east to west) and 5 km wide (from north to south) with an area of 30 km^2^. The ore body is laminated and exists in a set of shallowly metamorphosed muddy and sandy slates. The overall orientation of the ore body is north-west, which is basically consistent with the folded tectonic axis. The ore body trends to the north-east with a dip angle of 5°–55°, where the dip angle of ore body is 0°–30°. The morphology of some sections of the ore body has changed due to geological process, however, the ore body is stable along the strike and trend extension. The southeastern part of the Chambishi copper mine is the testing site of the National Key Research and Development Program of China under the project titled "Theory and Technology of Spatiotemporal Synergistically and Continuously Mining in a Multi-mining area of Deep Large Ore Section". The rock mass quality assessment of the Southeastern mine is a critical step to ensure the progress of several key technologies and the safety of industrial sites during the later stages of the project.

### Rock mass classification based GWO-SVC model

The current study evaluated the classification ability of three combined models and identified that the GWO-SVC exhibits the optimal classification ability. Herein, the GWO-SVC model is used to evaluate the rock mass quality in different areas of the southeastern ore zone of the Chambishi copper mine. During the assessment of rock mass quality, the following input parameters are determined, i.e., rock saturated compressive strength (R_c_), RQD value, integrity factor of the rock mass (K_v_), and unit roadway water inflow (ω). The investigators have conducted on-site surveys of the hanging wall and footwall, and characterized the rocks from the exposed southern and northern mining areas to ensure the accuracy of rock mass classification of the southeastern ore body. The accurate field values of RQD, K_v_, and ω of the rock mass were obtained through on-site borehole sampling, wave velocity testing and water seepage analysis. The mechanical strength testing capability in Zambia is insufficient. Therefore, we have transported the ore and rock specimens from each sampling area of the Southeastern ore body to the China to obtain comprehensive and reliable physical and mechanical parameters. The index parameters of each region are shown in Table 9.

**Table 9:** Southeast orebody rock data of chambishi copper.

Sample	R_c_/(MPa)	RQD%	K_v_	ω/[L (min 10 m)^−1^]
**Quartzite of hanging wall**				
Quartzite of hanging wall	96.86	52	0.45	25
Quartzite of hanging wall	151.63	64	0.65	1
Quartzite of footwall	172.61	67	0.65	1
Flint-bearing banded dolomite	56.49	68	0.65	20
**North mining area**				
Slate of ore body	127.92	72	0.65	2
Quartzite of hanging wall	98.23	74	0.65	10
Quartzite of footwall	81.06	80	0.65	0
Conglomerate of footwall	104.71	76	0.65	1
Base granite	162.36	65	0.65	0

Furthermore, GWO-SVC is used to predict the quality classification of typical rock masses in Southeastern orebody and the prediction results are shown in Table 10. The results reveal that the rock mass quality of the hanging and foot wall of the southeastern ore body in the south and north mining areas of the Chambishi copper mine is similar. The rock mass strength of the hanging wall and footwall in the southern mining area is better than the northern mining area, whereas the rock mass integrity of the southern mining area is weaker than the northern mining area. The orebody slate quality in the southern and northern mining areas is identified as Grade III and Grade II, which indicates that the quality of the same ore body exhibits little variation due to different rock-forming conditions and environments. Except for the slate of the orebody and flint-bearing banded dolomite in the southern mining area, which were wet, the rest of the samples are dry and less influenced by the groundwater. Overall, except for the slate of the orebody and flint-bearing banded dolomite in the southern mining area, which are evaluated as Grade III samples, the remaining samples belong to Grade II and the results reflect that the overall rock mass quality of the ore body in the southeastern region of the mine is highly stable and safe. Meanwhile, this study also applied traditional Rock Mass Rating (RMR) to classify the rock masses in the field and the classification results are shown in Table 10. The results obtained from RMR are consistent with the GWO-SVC results, further confirming the accuracy of GWO-SVC model.

**Table 10:** The comparison of GWO-SVC model prediction results and field RMR model classification results.

Sample	GWO-SVC	RMR
**The quartzite of hanging wall**		
The quartzite of hanging wall	III	III
The quartzite of hanging wall	II	II
Quartzite of footwall	II	II
Flint-bearing banded dolomite	III	III
**North mining area**		
The slate of ore body	II	III
The quartzite of hanging wall	II	III
Quartzite of footwall	II	II
Conglomerate of footwall	II	II
Base granite	II	II

## Limitation

By utilizing SVC as the predominant strategy to predict the rock mass quality, satisfactory prediction accuracies are procured. However, there are still some drawbacks and limitations that need to be improved in future work. Firstly, the scale of data used to establish the evaluation models is still small and only 80 groups of samples are collected. The combined model can learn more valid information when there are more samples from different sources in the dataset. Therefore, the dataset of the model should be further increased. Secondly, deeply analyzing rock characterization parameters is significant for rock mass classification. Thirdly, more advanced metaheuristic algorithms are worthwhile to be combined with SVC prediction models to improve the classification accuracy. For instance, the extreme gradient boosting^61^, are not investigated and compared in this study.

## Conclusions

The classification of rock mass is an important parameter for the design of underground engineering sites. The rock mass quality prediction and evaluation are always influenced by many factors. The relationship between these factors and the rock mass quality is elusive in different regions. Therefore it is difficult to grade the rock mass quality in different regions by some traditional method. AI-based techniques can simulate sophisticated relationships between influential factors and output targets compared to the conventional methods. In this study, we built a dataset containing 80 sets of samples, each containing four rock mass quality characterization parameters. To classify the rock masses, the SVC algorithm is used in this paper. Then, three types of optimal algorithms are combined with SVC to optimize the hyper-parameters. As a result, it is found that GWO-SVC obtains the most comprehensive classification performance. The accuracy of training and testing sets of GWO-SVC are 90.6250% (58/64) and 93.7500% (15/16), respectively. For Grades I, II, III, IV and V, the precision value is 1, 0.93, 0.90, 0.92, 0.83, the recall value is 1, 1, 0.93, 0.73, 0.83, and the F_1_ value is 1, 0.96, 0.92, 0.81, 0.83, respectively. According to the sensitivity analysis results, the RQD and Kv plays the most important role in influencing the rock mass quality. Finally, the GWO-SVC model, with optimal classification ability, is selected to classify the rock mass quality of the exposed area of southeastern ore body of the Chambishi copper mine in Zambia. The results reveal excellent consistency between GWO-SVC and RMR grading models, verifying the validity of GWO-SVC model for application in the field of rock mass.Therefore, the GWO-SVC rock mass classification model has good potential for application in the geotechnical field. After training with more data, the GWO-SVC model can become a powerful tool for engineering designers.
